# Shiga toxin 2 translocation across intestinal epithelium is linked to virulence of Shiga toxin-producing *Escherichia coli* in humans

**DOI:** 10.1099/mic.0.000645

**Published:** 2018-03-13

**Authors:** Seav-Ly Tran, Claire Jenkins, Valérie Livrelli, Stephanie Schüller

**Affiliations:** ^1^​ Norwich Medical School, University of East Anglia, Norwich, UK; ^2^​ Gut Health and Food Safety Programme, Quadram Institute Bioscience, Norwich, UK; ^3^​ Gastrointestinal Bacteria Reference Unit, Public Health England, London, UK; ^4^​ Université Clermont Auvergne, Inserm U1071, M2iSH 'Microbes, Intestin, Inflammation et Susceptibilité de l’Hôte', USC-INRA 2018, Clermont-Ferrand, France; ^5^​ CHU Clermont-Ferrand, Service de Bactériologie, Parasitologie Mycologie, Clermont-Ferrand, France; ^†^​ Present address: Micalis Institute, INRA, AgroParisTech, Université Paris-Saclay, Jouy-en-Josas, France.

**Keywords:** STEC, Shiga toxin, HUS, human intestinal epithelium, seropathotype

## Abstract

Shiga toxin-producing *Escherichia coli* (STEC) are characterized by the release of potent Shiga toxins (Stx), which are associated with severe intestinal and renal disease. Although all STEC strains produce Stx, only a few serotypes cause infection in humans. To determine which virulence traits *in vitro* are linked to human disease *in vivo*, 13 Stx2a-producing STEC strains of seropathotype (SPT) A or B (associated with severe human intestinal disease and outbreaks) and 6 strains of SPT D or E (rarely or not linked to human disease) were evaluated in a microaerobic human colonic epithelial infection model. All SPT strains demonstrated similar growth, colonization of polarized T84 colon carcinoma cells and Stx release into the medium. In contrast, Stx translocation across the T84 cell monolayer was significantly lower in SPT group DE compared to SPT group AB strains. Further experiments showed that Stx penetration occurred via a transcellular pathway and was independent of bacterial type III secretion and attaching and effacing lesion formation. These results suggest that the extent of Stx transcytosis across the gut epithelium may represent an important indicator of STEC pathogenicity for humans.

## Introduction

Shiga toxin-producing *E. coli* (STEC) are major foodborne pathogens in the developed world and cause around 1000 reported infections per year in the UK [1, 2]. Apart from gastroenteritis, persons infected with STEC can develop haemorrhagic colitis and haemolytic uraemic syndrome (HUS), a severe systemic disease associated with renal failure and neurological damage [3, 4]. STEC have their natural reservoir in the gastrointestinal tract of ruminants, particularly cattle. Therefore, most infections are linked to consumption of undercooked beef, contaminated fresh produce or direct contact with ruminant animals [5].

STEC strains can have many different serotypes, but some are more closely associated with disease than others. In 2003, Karmali and colleagues introduced a graded classification system of five seropathotypes (SPTs A to E) based on the association of serotypes with human intestinal disease, outbreaks and HUS [6]. In this system, SPT A comprises serotype O157 : H7, which is most commonly associated with human disease, followed by SPT B, which includes non-O157 STEC linked to outbreaks and HUS (e.g. O26, O45, O103, O111, O121 and O145 – also known as the ‘big six’). On the other end of the spectrum, SPT D and E are rarely or never found in humans and do not cause severe disease [6].

In accordance with STEC pathogenesis, SPT A and B strains generally harbour the locus of enterocyte effacement (LEE) pathogenicity island, which encodes a type III secretion system (T3SS), the outer-membrane protein intimin, the translocated intimin receptor (Tir) and associated effector proteins [7]. After establishing initial contact with human intestinal epithelium via fimbriae, pili or flagella, STEC express a filamentous T3SS and inject their own receptor Tir into the host cell, where it becomes inserted in the cytoplasmic membrane [8, 9]. Subsequent binding of intimin to Tir allows STEC to adhere tightly to the epithelium by forming an attaching and effacing lesion [10]. In addition to Tir, other bacterial effector proteins are injected into the host cell, and these induce epithelial permeability and changes in ion absorption and inflammation, and subsequently lead to the development of diarrhoea [11]. In addition to the T3SS, STEC pathogenicity is dependent on the production of Shiga toxins (Stx), which are required for the development of HUS. Stx are highly cytotoxic to cells expressing globotriaosylceramide (Gb3) on their surface, such as the microvasculature in the intestine, kidney and brain [12]. STEC can express Stx1 or Stx2, which are antigenically different [13]. These can be further divided into several subtypes, of which Stx2a has been most strongly associated with HUS [14, 15]. To cause systemic disease, Stx must penetrate the intestinal epithelium, which does not express Gb3 [16, 17]. How this happens during STEC infection remains unknown, but *in vitro* studies using human intestinal epithelial cell lines have demonstrated both paracellular and transcellular Stx transport [18–20].

In our previous work, we investigated the effect of oxygen on STEC pathogenesis by using a vertical diffusion chamber (VDC) and showed that low oxygen levels similar to those in the colon promote bacterial colonization and Stx transcytosis across polarized human intestinal epithelial cells [20, 21]. In this study, we investigated whether the potential of STEC to cause severe disease in humans was associated with certain virulence traits *in vitro*. To this aim, we selected 13 Stx2a-expressing STEC strains from SPT A and B (high association with human disease) and 6 strains from SPT D and E (low/no association with human disease) and evaluated bacterial growth, epithelial colonization and barrier function, and Stx production and translocation in a microaerobic human intestinal VDC infection model.

## Methods

### Bacterial strains

The STEC wild-type strains used in this study are listed in Table 1 and have been classified into seropathotypes according to the system described by Karmali and colleagues [6]. For each strain, Stx type and presence of the LEE was determined by PCR and colony blot hybridization as described previously [22–25]. The EDL933 deletion mutants in *eae* and *espA* were kindly provided by Grégory Jubelin (INRA, Clermont-Ferrand, France) and Carlos Guzmán (Helmholtz Centre for Infection Research, Braunschweig, Germany), respectively, and have been described previously [26, 27]. Bacteria were grown standing in Luria–Bertani (LB) broth overnight at 37 °C. Strain EDL933 Δ*eae* was selected with kanamycin (50 µg ml^−1^). Bacteria were spun down before infection and suspended in serum-free culture medium.

**Table 1.** STEC strains used in this study

SPT	Code	Designation	Serotype	Origin*	Stx	LEE	Reference/source†
A	A	H122900261	O157 : H7	H	2a	+	PHE
A	B	H122280235	O157 : H7	H	2a	+	PHE
A	C	H121800418	O157 : H7	H	2a	+	PHE
A	D	H093740759	O157 : H7	H	2a, 2c	+	PHE
A	E	H122800318	O157 : H7	H	2a	+	PHE
A	F	H121800416	O157 : H7	H	2a	+	PHE
A	G	Walla-1	O157 : H7	H	2a	+	[39]
A	H	EDL933	O157 : H7	H	1a, 2a	+	[40]
A	I	H071840336	O157 : H7	H	2a, 2c	+	PHE
B	J	E135309	O26 : H11	H	2a	+	PHE
B	K	H103540554	O145 : H28	H	2a	+	PHE
B	L	H10466046701	O26 : H11	H	2a	+	PHE
B	M	H12316062601	O26 : H11	H	2a	+	PHE
D	N	NV283	O6 : H+	B	2a	−	M2iSH
D	O	NV303	O77 : H18	B	2a	−	M2iSH
D	P	NV196	O116 : H21	B	2a	−	M2iSH
E	Q	NV282	O96 : H19	B	2a	−	M2iSH
E	R	E154625	O2 : H21	B	2a	−	PHE
E	S	E150972	O2 : H26	B	2a	−	PHE

*Human (H) or bovine (B) origin.

†PHE, Public Health England, London, UK; M2iSH, ‘Microbes, Intestin, Inflammation et Susceptibilité de l’Hôte’, USC-INRA 2018, Clermont-Ferrand, France.

### Cell culture

Human T84 colon carcinoma cells (ATCC CCL-248) were cultured in Dulbecco’s modified Eagle’s medium/F-12 mixture supplemented with 10 % foetal calf serum (FCS; Sigma) and used between passages 42 and 65. For vertical diffusion chamber experiments, 5×10^5^ T84 cells/cm^2^ were seeded on collagen-coated Snapwell filter inserts (12 mm diameter, 0.4 µm pore; Corning Costar). Cells were differentiated for 12–18 days until transepithelial electrical resistance (TEER) reached 1000 to 2500 Ω × cm^2^. TEER was monitored using an EndOhm chamber and EVOM resistance meter (WPI). African Green monkey kidney Vero cells (ECACC 84113001) were cultured in Dulbecco’s modified Eagle’s medium supplemented with 10 % FCS. All cells were grown at 37 °C in a 5 % CO_2_ atmosphere.

### Microaerobic infection in a vertical diffusion chamber

VDC experiments were performed as described previously [20, 21]. Briefly, polarized T84 cells were infected with 5×10^5^ to 5×10^6^ bacteria on the apical side. Apical chambers were perfused with an anaerobic gas mixture (90 % N_2_, 5 % H_2_, 5 % CO_2_), while basal compartments were kept under aerobic conditions (5 % CO_2_ in air). The oxygen concentrations in apical compartments reached 1.4–1.7 % of atmospheric pressure, as determined with an ISO2 dissolved oxygen meter (WPI). Incubations were performed for 5 h.

### Stx quantification by Vero cell cytotoxicity assay

Bacteria were removed from apical and basal media by centrifugation (apical media only) and subsequent filter sterilization. Apical samples were diluted 1 : 15, and basal samples were concentrated 25-fold with Amicon Ultra centrifugal filter units (Millipore). Stx concentrations were determined by Vero cell cytotoxicity assay [20]. Briefly, 50 µl of sample or diluted purified Stx2a as standard (0.01–1000 ng ml^−1^; Anne Kane, Tufts Medical Center, Boston, USA) were pipetted into a 96-well cell culture plate. Samples were tested in duplicate and standards in triplicate wells. Vero cells were added to each sample (2×10^4^ cells in 100 µl) and plates were incubated for 72 h at 37 °C in a 5 % CO_2_ atmosphere. Cells were washed with PBS, fixed in 3.7 % formaldehyde in PBS and stained with 0.1 % (w/v) crystal violet in 10 % ethanol for 20 min. After elution of the dye in 50 % ethanol, crystal violet staining was quantified by optical density at 595 nm (Benchmark Plus, Bio Rad). Stx sample concentrations were calculated from Stx standard curves using Microplate Manager 5.2.1 software (Bio Rad). When comparing Stx release between strains, Stx concentrations were adjusted according to the optical density at 600 nm (OD_600_) of the apical media to account for differences in bacterial growth. Stx translocation was calculated as follows: (Stx concentration in basal medium/Stx concentration in apical medium)×100 and expressed as a percentage.

### Stx quantification by ELISA

Stx2 in apical supernatants was quantified using a sandwich ELISA as described previously [28] with the following modifications. Microtitre plates were coated with rabbit polyclonal anti-Stx2 (rb204, 1 : 5000; kindly provided by Anne Kane, Tufts Medical Center, Boston, USA) and Stx2 was detected using mouse monoclonal antibody NR-846 (1 : 2500; Biodefense and Emerging Infections Research Resources Repository, NIAID, NIH, USA).

### Quantification of STEC colonization

Snapwell inserts were removed from the VDC and washed twice in sterile PBS to remove non-adherent bacteria. After lysis of cell monolayers in 1 % Triton X-100 in PBS for 20 min, serial dilutions were prepared and plated out on LB agar plates. Plates were incubated at 37 °C overnight and colony-forming units were counted.

### Statistics

Statistical analysis was performed using GraphPad Prism software (version 6). Student’s unpaired *t*-test was used to determine differences between two groups. One-way ANOVA with Tukey’s or Dunnett’s multiple comparisons test was used to analyse differences between multiple groups. A *P*-value of <0.05 was considered significant. The data are shown as means±standard errors of the mean.

## Results

### Growth and colonization of polarized T84 cells by STEC strains of different SPTs

**Fig. 1.**
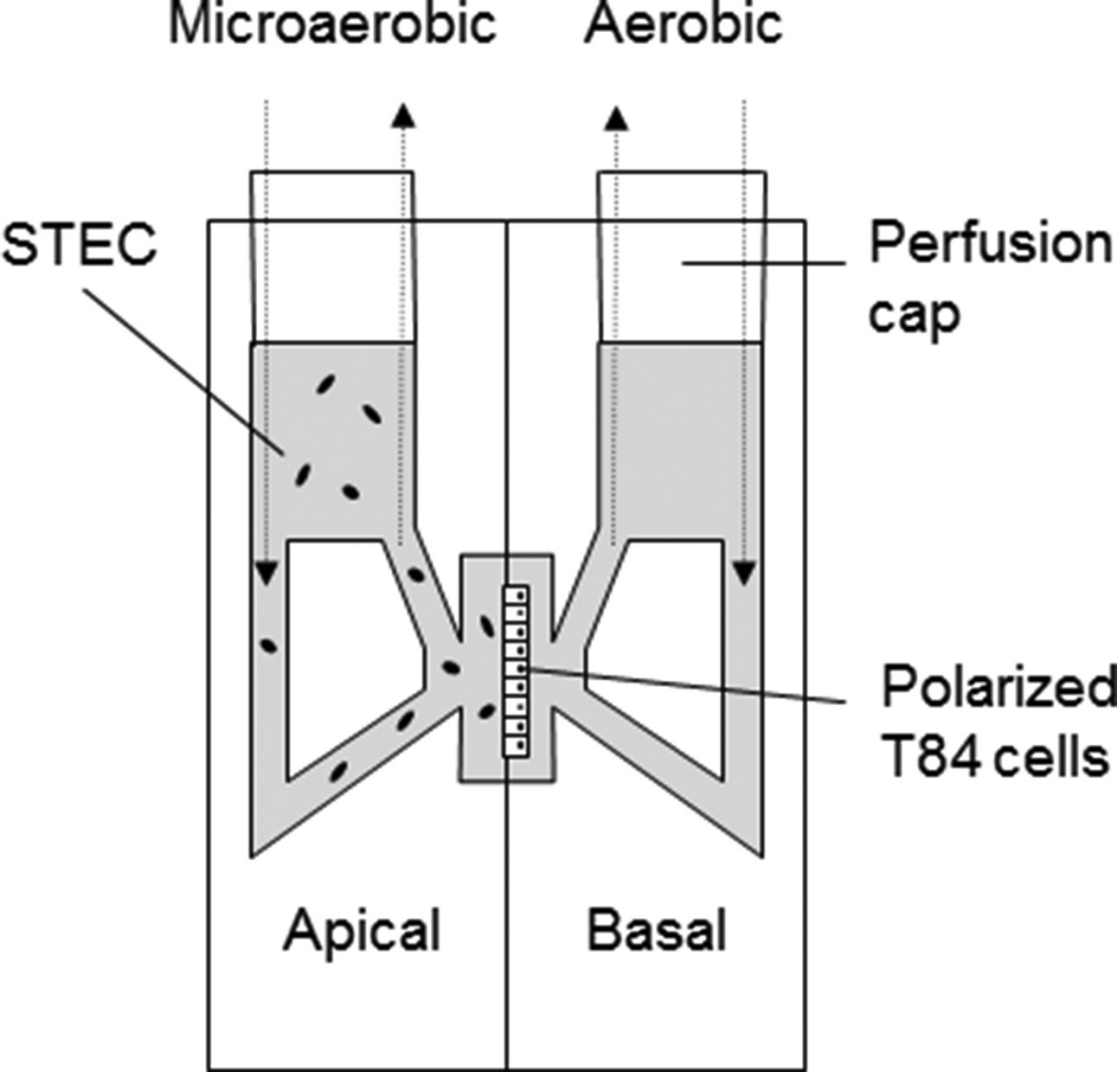
Schematic representation of vertical diffusion chamber. Polarized T84 cells grown on Snapwell filters were inserted between two half-chambers and infected apically with STEC. Apical chambers were maintained under microaerobic conditions, whereas basal compartments were kept under aerobic conditions.

**Fig. 2.**
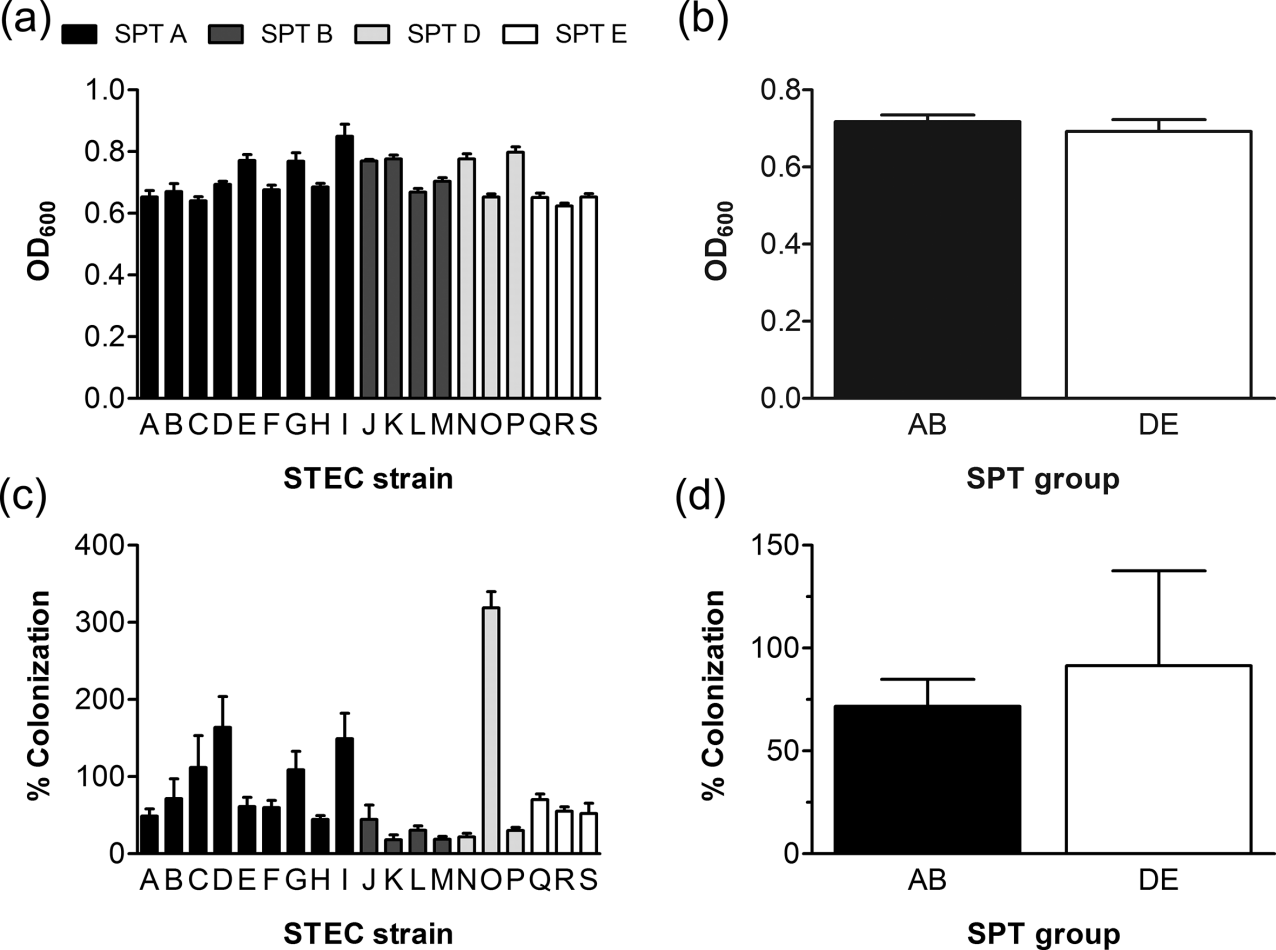
Growth and colonization of STEC SPT strains during VDC infection. T84 cells were infected with SPT strains for 5 h. (a, b) Growth of non-adherent bacteria was determined by OD_600_. (c, d) Adherent bacteria were quantified by plating out serial dilutions of cell lysates and counting c.f.u.s. Colonization is expressed as percentage of cell-associated bacteria relative to the inoculum. Results are shown for individual strains (a, c) and for SPT groups AB and DE combined (b, d). *n*=5 in triplicate.

To determine whether higher virulence of SPT AB versus DE strains was related to better growth and colonization of human intestinal epithelium, infections of polarized T84 cells were carried out in a microaerobic VDC system for 5 h (Fig. 1). Growth of non-adherent bacteria was determined by measuring the OD_600_ of apical media, whereas colonization of polarized epithelia was quantified by plating out serial dilutions of cell lysates and counting c.f.u.s. As shown in Fig. 2(a, b), all strains showed similar growth during incubation in the VDC. In contrast, the colonization of polarized T84 cells differed among strains, with large variations within both SPT groups (Fig. 2c). No significant differences were detected between SPT groups AB and DE (Fig. 2d).

### Association of SPT with Stx production and translocation

**Fig. 3.**
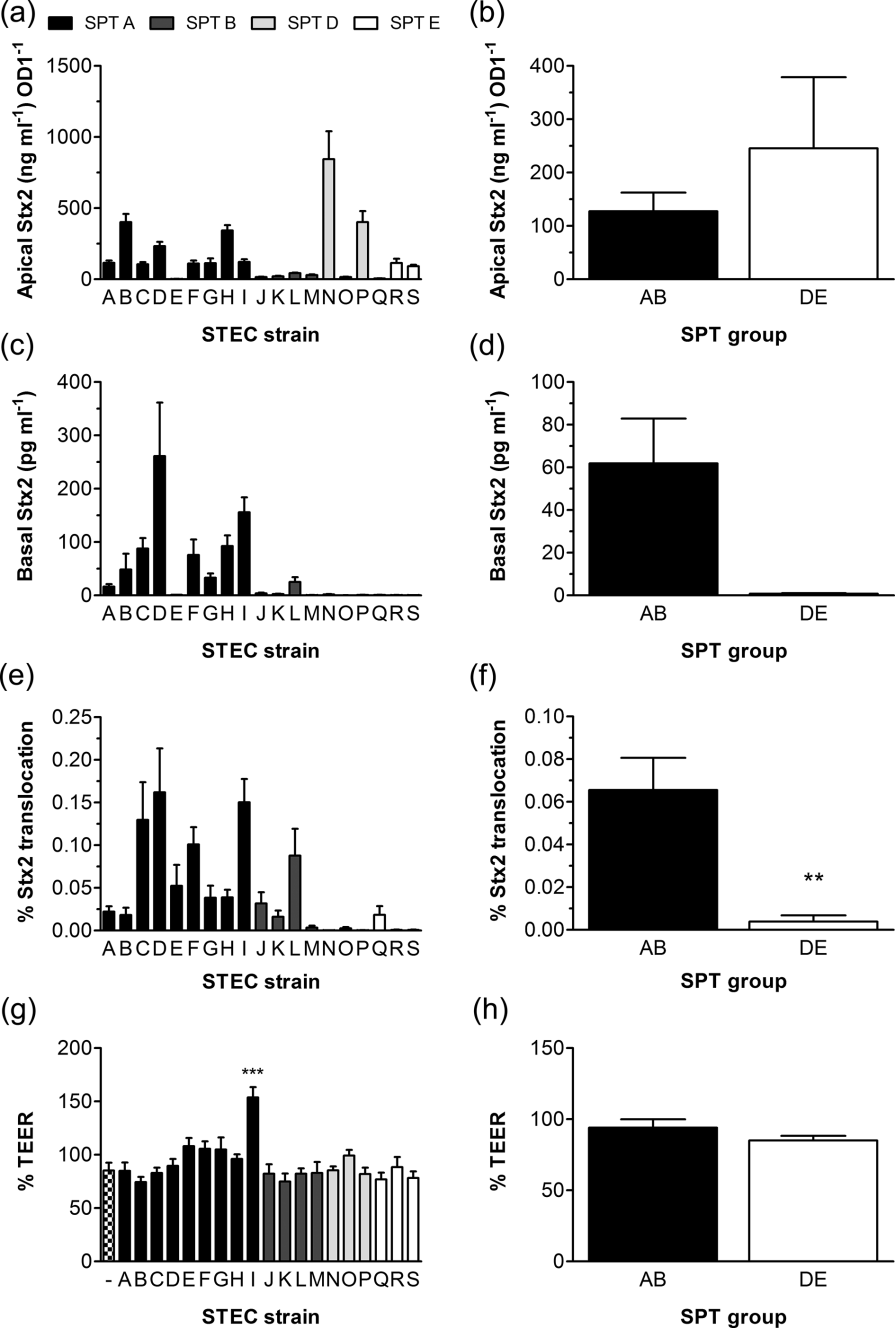
SPT Stx2 release and translocation across the epithelial monolayer. T84 cells were infected with SPT strains for 5 h or left non-infected (−). Stx2 concentrations in apical and basal media were determined by Vero cell assay. (a, b) Stx2 levels in apical media were normalized to an OD_600_ of 1.0. (c, d) Stx2 levels in basal media. (e, f) Stx2 translocation is expressed as percentage of apical Stx recovered in basal compartments. (g, h) TEER after infection is expressed relative to TEER before infection. Results are shown for individual strains (a, c, e, g) and for SPT groups AB and DE combined (b, d, f, h). *n*=5 in triplicate. **, *P*<0.01 vs SPT AB; ***, *P*<0.001 vs non-infected control.

We further investigated the correlation of SPT with Stx production during infection. Stx release into the apical medium was quantified by Vero cell cytotoxicity assay (Fig. 3a, b, Table S1, available in the online version of this article). Similar to colonization, Stx production was variable among SPT strains and, taken together, there was no significant difference between SPT groups AB and DE (Fig. 3a, b). Stx concentrations in apical media were also determined by ELISA for a selected set of samples, and the results showed that both detection methods exhibited a similar trend for all strains, but the Vero cell assay demonstrated a greater dynamic range compared to ELISA (Fig. S1). In addition to Stx release, we evaluated Stx penetration across the epithelial cell layer by quantifying Stx levels in basal compartments. This was performed by Vero cell assay, as ELISA was not sensitive enough. Whereas the SPT A and B strains demonstrated variable levels of Stx in basal media, the levels were consistently low in the SPT D and E strains (Fig. 3c, d, Table S1). The calculation of Stx penetration relative to amounts of toxin released into apical media indicated significantly lower percentages of translocated Stx during infection with SPT group DE vs group AB strains (Fig. 3e, f). As Stx can penetrate the epithelium paracellularly or transcellularly, we also determined epithelial barrier function by measuring the TEER of the T84 monolayers before and after infection. Non-infected epithelia were included as controls. Whereas none of the strains significantly disrupted epithelial barrier function, infection with strain I (SPT A) resulted in increased monolayer resistance versus the non-infected control (Fig. 3g). Overall, the strains of SPT groups AB and DE did not significantly differ in affecting epithelial permeability (Fig. 3h).

### Influence of type III secretion and intimin on Stx translocation

**Fig. 4.**
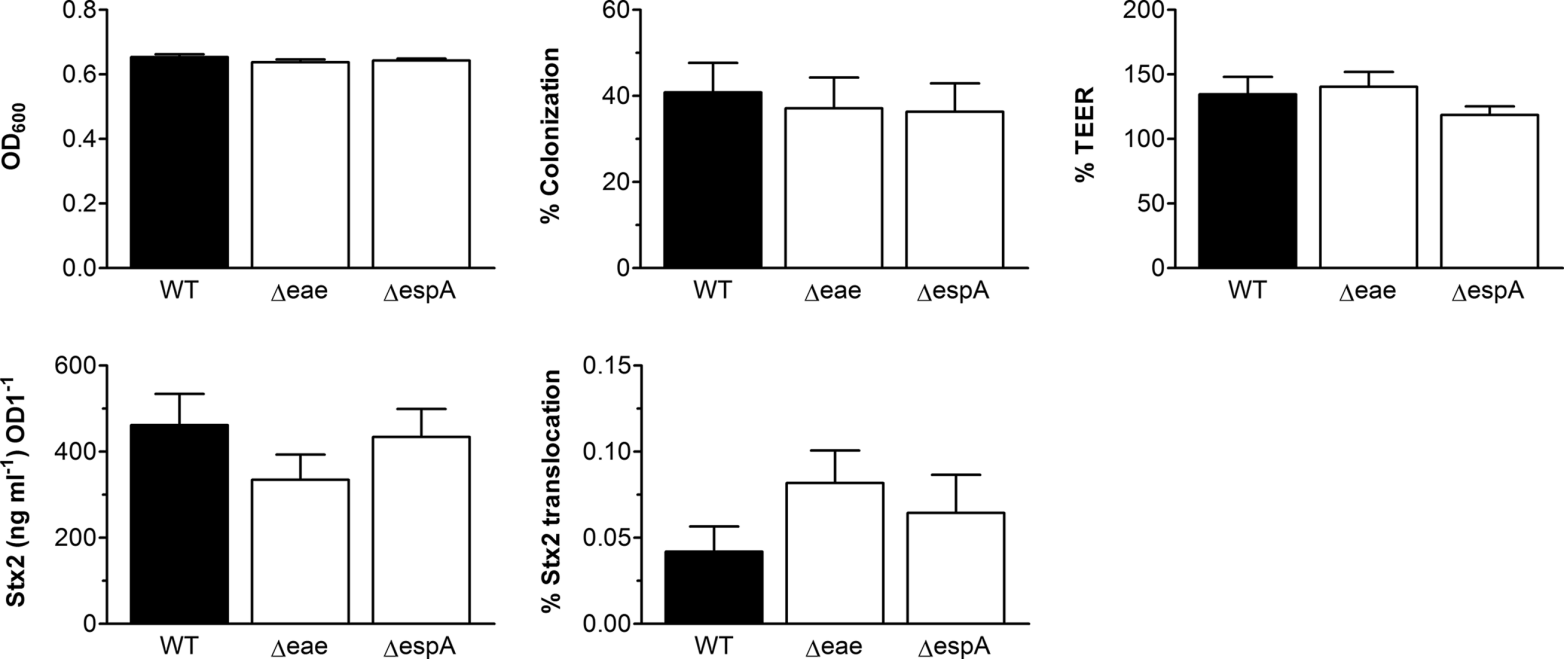
STEC Stx2 translocation is not dependent on intimin or T3S. T84 cells were infected with SPT A wild-type strain EDL933 (WT) or isogenic deletion mutants in intimin (Δeae) or EspA (ΔespA) for 5 h. Bacterial growth, colonization, epithelial barrier function, Stx release and translocation were determined as described in Fig 2 and 3. *n*=6 in triplicate.

As all strains of SPT group AB, but not those of group DE, contain the LEE and therefore express the T3SS and intimin, we investigated whether any of these factors were responsible for enhanced Stx translocation by SPT group AB versus DE. VDC infections were performed with SPT A strain EDL933 and isogenic deletion mutants in *eae* (intimin) and *espA* (T3SS translocation filament). As shown in Fig. 4, no significant differences in bacterial growth, colonization, epithelial permeability, Stx production and translocation were observed in infections with the mutants versus the wild-type strain.

## Discussion

STEC are defined by the production of Shiga toxins, but this alone is not sufficient to cause human disease. Epidemiological studies have linked certain serotypes to human pathogenicity, and this is the basis for the SPT classification introduced by Karmali and colleagues [6]. While all of the SPT AB strains used in this study are human clinical isolates, all of the SPT DE strains are of bovine origin due to their rare association with human disease. Therefore, their potential virulence in humans can only be inferred based on epidemiological trends. In an attempt to define what makes STEC pathogenic, SPT strains have been analysed for their virulence gene profiles, and *eae* encoding intimin as part of the LEE has been identified as a strong virulence indicator [6]. Furthermore, genes for putative adhesins, *toxB* and *efa1*, were associated with SPT A and B, while long polar fimbriae (*lpfA*_O157/OI-141_ and *lpfA*_O157/OI-154_) were more frequently found in SPT A [29]. Despite genotypic differences in adhesins between SPTs, we did not detect any significant difference in the colonization of polarized T84 cells by SPT AB and SPT DE strains. This agrees with a previous study showing poor correlation of SPT and *in vitro* adherence to HEp-2 and HCT-8 cell lines. The colonization levels were generally low and SPT B showed the highest binding, but some SPT DE strains also adhered well [30]. Given the presence of the LEE and intimin gene in SPT AB but not SPT DE strains, this is surprising. However, we demonstrated earlier that STEC O157 : H7 do not translocate Tir into polarized T84 cells and that binding occurs independently of intimin in this cell model [10]. This is also confirmed in this study, where an O157 intimin mutant showed similar colonization to the corresponding wild-type strain.

In addition to colonization of the intestinal epithelium, Stx production is required for causing disease. Previous studies have demonstrated that *stx2a* and *stx2d* are more closely associated with SPT AB than SPT DE [25]. Notably, we identified far fewer Stx2a-expressing SPT DE than SPT AB strains for this study, and only 2 Stx2a-positive isolates were found amongst a collection of 49 Stx2-producing bovine isolates at Public Health England. As Stx types and subtypes demonstrate differences in cytotoxicity [13, 31], we aimed to select strains expressing Stx2a only. EDL933 was included as the O157 : H7 prototype strain, and this strain also produces Stx1a. However, previous studies in our group have shown that Stx2a is the major toxin type released during EDL933 infection, whereas Stx1a remains in the periplasm [20]. SPT A strains D and I also produce Stx2c. As purified Stx2a has been shown to be at least 25 times more toxic in Vero cells than Stx2c [31], this is unlikely to considerably influence total Stx production by these strains. In this study, we did not detect any correlation between Stx2 release and SPT group. Previous reports have shown higher Stx2 production by human- versus bovine-derived isolates [32, 33], but no SPT classification was performed in the first study [33], whereas both human and bovine strains belonged to SPT A and C in the second study [32]. In addition, Stx production was determined in LB broth cultures in the absence of host cells. In contrast to Stx release, we observed a significantly higher translocation of Stx2 across the epithelial monolayer during infection with SPT AB versus SPT DE strains. As epithelial resistance was not compromised during infection, Stx is likely transported by a transcellular rather than a paracellular pathway. Stx transcytosis across polarized T84 cells can be mediated by macropinocytosis or independent mechanisms and is enhanced by STEC infection [20, 34]. Here, we demonstrate that Stx translocation during infection with prototype strain EDL933 does not require T3S or intimin, but this needs to be confirmed for other SPT AB strains. There appear to be other factors present in SPT AB but not SPT DE strains promoting Stx penetration of intestinal epithelium. One candidate is the secreted serine protease EspP, which has been shown to stimulate host actin remodelling and Stx macropinocytosis [34]. While a similar frequency of *espP* was detected in STEC isolated from humans, cases of severe disease and animals/food [14], a recent study showed an association of *espP* with 100 % of SPT AB strains, but only 41 % of SPT DE strains [30]. In addition, the putative pathogenicity islands OI-57, OI-71 and OI-122 harbouring genes for non-LEE encoded T3S effectors (*nle*) appear to be more prevalent in SPT A and B than in other SPTs [6, 35–37]. In fact, the genes *nleA*, *nleB*, *nleC*, *nleE*, *nleF*, *nleG*, *nleG2-1*, *nleG2-3*, *nleG5-2*, *nleG6-2*, *nleG9*, *nleH1*, *nleH2* and *ent/espL2* were more prevalent in isolates associated with HUS (SPT A–C) than in strains associated with only diarrhoea or animals (SPT DE) [37]. Nle effector proteins have been shown to inhibit host cell apoptosis and inflammatory response [38], but no involvement in Stx uptake and transport across the epithelium has been established yet.

In this study, we report an association between SPT and Stx translocation across the intestinal epithelium, with higher levels of Stx2a transcytosis being observed during infection with STEC SPT group AB versus SPT group DE strains. Future studies will focus on the underlying molecular mechanisms and genetic determinants and should lead to the identification of genetic markers for highly virulent STEC strains.

## Supplementary Data

256 Supplementary File 1
