# Supplementary material for: Shiga toxin 2 translocation across intestinal epithelium is linked to virulence of Shiga toxin-producing Escherichia coli in humans
Source: Microbiology (Reading). 2018 Mar 13;164(4):509–16. doi: 10.1099/mic.0.000645 (PMC5982136; doi:10.1099/mic.0.000645)

Table S1: Apical and basal Stx2 concentrations determined by Vero cell assay and calculated translocation rates

	Apical Stx2 (ng/ml)[a]	OD_{600}	Apical Stx2/ OD1 (ng/ml)[b]	Basal Stx2 conc (ng/ml)[c]	% Stx2 translocation[d]
A	75.1 ± 8.1	0.65 ± 0.02	115.5 ± 15.9	0.413 ± 0.114	0.022 ± 0.006
B	269.3 ± 31.9	0.67 ± 0.03	402.0 ± 57.6	1.212 ± 0.742	0.018 ± 0.008
C	67.5 ± 11.9	0.64 ± 0.01	105.5 ± 15.2	2.194 ± 0.490	0.130 ± 0.044
D	161.2 ± 24.6	0.69 ± 0.01	233.6 ± 29.7	6.529 ± 2.507	0.162 ± 0.051
E	1.9 ± 0.4	0.77 ± 0.02	2.5 ± 0.5	0.025 ± 0.008	0.052 ± 0.025
F	75.2 ± 18.7	0.68 ± 0.01	110.6 ± 21.4	1.899 ± 0.722	0.101 ± 0.020
G	87.1 ± 23.9	0.77 ± 0.03	113.1 ± 34.2	0.827 ± 0.202	0.038 ± 0.014
H	237.0 ± 30.1	0.69 ± 0.01	343.5 ± 37.5	2.311 ± 0.502	0.039 ± 0.009
I	103.8 ± 14.9	0.85 ± 0.04	122.1 ± 18.9	3.893 ± 0.705	0.150 ± 0.027
J	11.9 ± 1.7	0.77 ± 0.01	15.4 ± 2.2	0.095 ± 0.033	0.032 ± 0.013
K	16.5 ± 2.2	0.78 ± 0.01	21.1 ± 2.7	0.066 ± 0.011	0.016 ± 0.007
L	28.5 ± 3.8	0.67 ± 0.01	42.5 ± 4.8	0.627 ± 0.230	0.088 ± 0.031
M	20.5 ± 3.2	0.70 ± 0.01	29.3 ± 5.2	0.015 ± 0.003	0.003 ± 0.002
N	658.2 ± 150.2	0.78 ± 0.02	843.8 ± 196.5	0.049 ± 0.012	0.0003 ± 0.0001
O	10.3 ± 1.9	0.65 ± 0.01	15.8 ± 2.9	0.008 ± 0.003	0.003 ± 0.001
P	321.8 ± 56.1	0.80 ± 0.02	402.3 ± 77.7	0.024 ± 0.006	0.0003 ± 0.0001
Q	3.8 ± 0.6	0.65 ± 0.01	5.9 ± 1.1	0.018 ± 0.009	0.019 ± 0.010
R	70.7 ± 8.9	0.62 ± 0.01	114.1 ± 30.3	0.012 ± 0.006	0.0007 ± 0.0003
S	59.6 ± 8.2	0.65 ± 0.01	91.7 ± 10.4	0.010 ± 0.006	0.0007 ± 0.0003

[a] Stx2 concentrations in apical supernatants [Stx_{apical}]

[b] Apical Stx2 concentrations adjusted to OD1 calculated as [Stx_{apical}]/OD_{600}

[c] Stx2 concentrations in 25fold concentrated basal supernatants [Stx_{basal}]

[d] Percentage Stx2 translocation calculated as $\frac{[\text{Stx basal}]/25}{[\text{Stx apical}]}$ x 100

Figure S1. Stx concentrations in apical media determined by ELISA and Vero cell assay (VCCA, n=3).

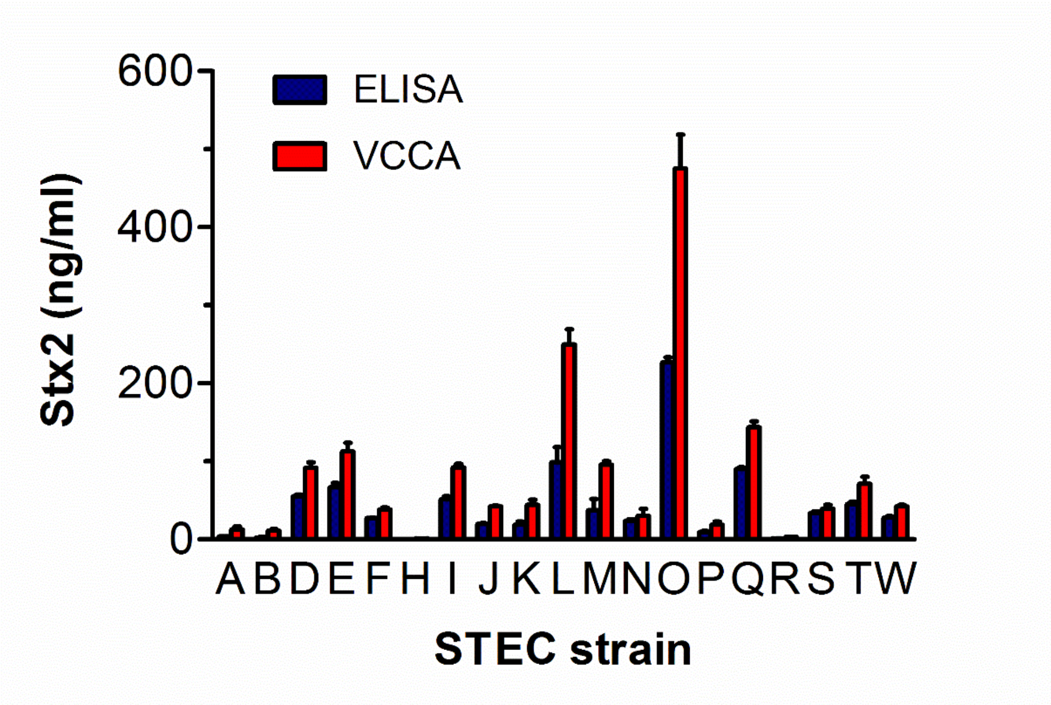

Supplement: Supplementary File 1 [file mic-164-509-s001.pdf]
